# Biomass acid-catalyzed liquefaction – Catalysts performance and polyhydric alcohol influence

**DOI:** 10.1016/j.dib.2015.10.037

**Published:** 2015-11-06

**Authors:** Maria Margarida Mateus, Ricardo Carvalho, João Carlos Bordado, Rui Galhano dos Santos

**Affiliations:** CERENA, Departamento de Engenharia Química e Biológica, Torre Sul, Instituto Superior Técnico, Av. Rovisco Pais, 1049-001 Lisboa, Portugal

**Keywords:** Liquefaction, Catalysts, Solvents, Biooils, cork

## Abstract

Herein, the data acquired regarding the preliminary experiments conducted with different catalyst, as well as with two polyhydric alcohols (glycerol and 2-ethylhexanol), for the preparation biooils from cork liquefaction at 160 °C, is disclosed. This data may be helpful for those who intent to outline a liquefaction procedure avoiding, thus, high number of experiments.

## Specifications Table

**Table:** 

Subject area	*Chemistry*
More specific subject area	*Chemical Engineering*
Type of data	*figure*
How data was acquired	*Conversion yield was determined based on solid residue content*
Data format	*analyzed*
Experimental factors	*The samples were subjected to moderate temperatures in the presence of a acid catalyst and polyhydric alcohols without pre-treatment*
Experimental features	*Thermochemical liquefaction of cork catalyzed by acids*
Data source location	*Lisbon, Portugal, GPS: 38° 44׳ 10.31׳׳N; 9° 08׳ 19.66׳׳W*
Data accessibility	*Data is provided in the article*

## Value of the data

**Table:** 

•	The assembled data regards the performance of different catalyst during the liquefaction of cork.
•	Comparison between a mineral, organic and a Lewis acid.
•	The influence of two different *polyhydric alcohols* was screened.

## 1 Experimental design, materials and methods

### 1.1 Materials and chemicals

Cork Supply SA kindly supplied Cork powder. The reagents used were chemical grade and purchased from Sigma-Aldrich.

### 1.2 Liquefaction procedure

The adopted procedure for the liquefaction of cork was as described by Mateus et al. [1]: the reaction vessels were loaded with a mixture of solvents with a ratio of 1/2 w/w of polyhydric alcohol (glycerol or 2-ethylhexanol) and diethylene glycol (DEG) ratio, containing a 3% or 1.5% of catalyst [sulfuric acid (H_2_SO_4_), p-Toluenesulfonic acid (*p*-TsOH) and Praseodymium(III) trifluoromethanesulfonate (Pr(OTf)_3_)] 10% w/w of cork powder. The reaction mixture was heated and the temperature controlled at 160 °C. The reaction was stopped when the conversion was higher than 95%. Afterwards the vessels were allowed to cool to room temperature. During the liquefaction process, samples were regularly retrieved to evaluate the liquefaction yield.

### 1.3 Measurement of liquefaction extent

The conversion was gravimetrically evaluated based on the residue content (unreacted raw material). A sample of the reaction mixture was diluted with acetone and filtered Afterwards the residual solid was washed with acetone and then dried in an oven set to 120 °C until constant weight. The liquefaction yield was calculated by the following equation:

(1) $$\text{Liquefaction}\;\text{yield}\;\left(\%\right)=\left(1-\frac{M_{2}\times M_{m}}{M_{S}\times M_{1}}\right)\times 100$$

where *M*_1_ is the initial mass of cork, *M*_2_ the mass of the residue obtained, *M_s_* the weight of the sample withdrawn and the *M_m_* is the initial mass of the reaction mixture.

## 2 Data analysis

**Fig. 1:**
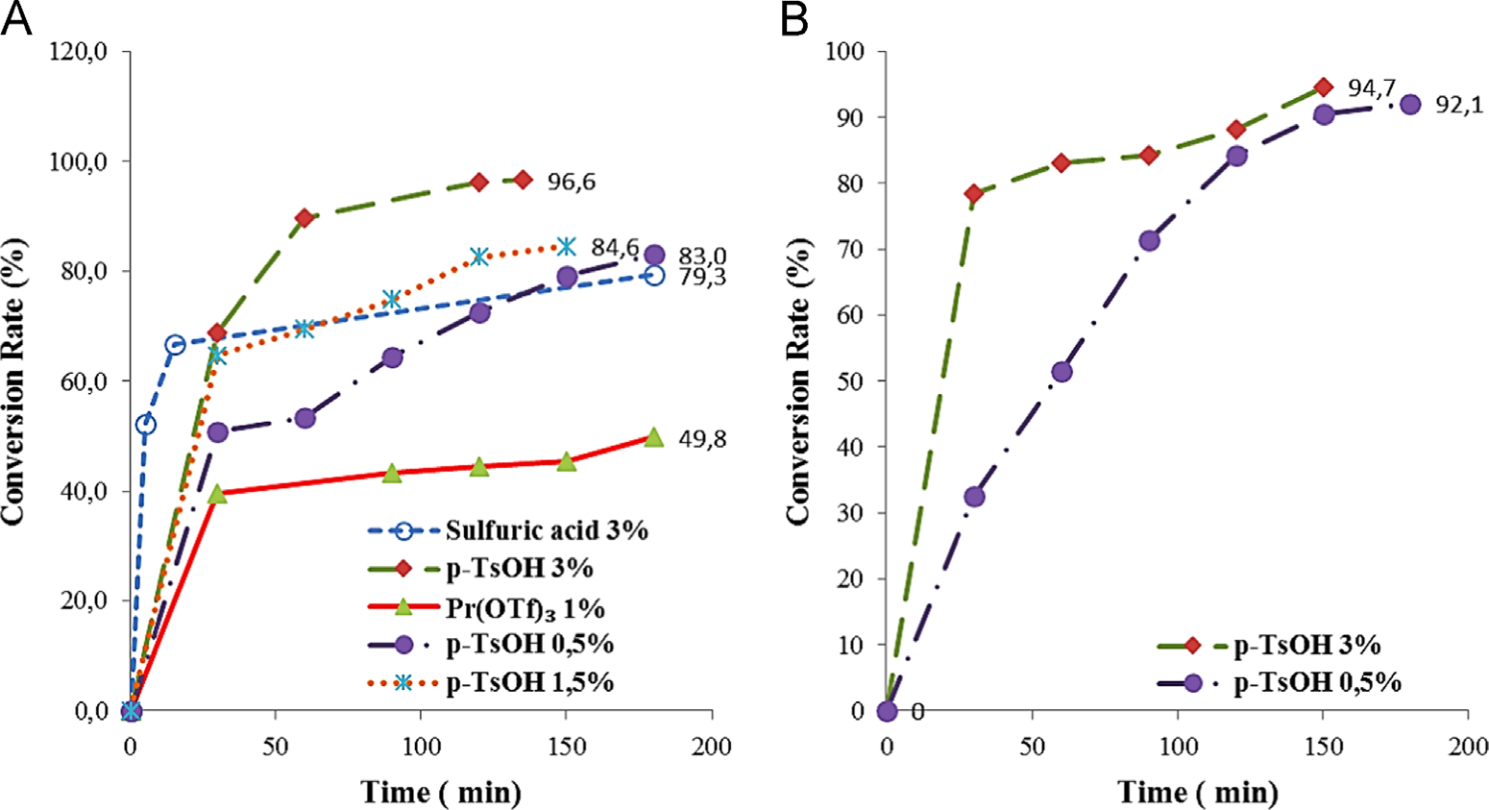
Liquefaction of cork at 160 °C in: (A) glycerol/DEG and (B) 2-ethylhexanol/DEG.

The data acquired is analyzed and plotted in Fig. 1.
